# Miquel Porta’s seventh edition of the Dictionary of Epidemiology

**DOI:** 10.1093/ije/dyag148

**Published:** 2026-08-19

**Authors:** Cesar Victora

**Affiliations:** Emeritus Professor, Post-Graduate Programme in Epidemiology, Universidade Federal de Pelotas, Pelotas, RS, Brazil

As someone who became an epidemiologist in the pre-Internet era, one of the essential half a dozen books on my office’s front shelf was the first edition of the Dictionary of Epidemiology, currently in its seventh edition (Porta M. A Dictionary of Epidemiology (7th Edition). Oxford, UK / New York, NY: Oxford University Press, 2026, 384 pages, ISBN: 9780197663622 (Hardcover) / 9780197663639 (Paperback)). This 1983 edition was a pocketbook with about 1000 entries in just over 100 pages, which I received as a gift when joining the International Epidemiological Association. I later learned from its founding editor, the late John Last, that he designed it as a “checkbook-sized” handbook that field researchers, medical students, and clinical workers could easily carry in a pocket or small bag. While we no longer carry printed books or even checkbooks around, the dictionary remains an essential tool for epidemiologists.

In just over four decades, its seventh edition grew to more than 2500 entries across almost 400 pages. Since the fifth edition, the printed version no longer fits in a pocket, but the e-book version is readily accessible through smartphones. The history of these seven editions reflects how rapidly epidemiology expanded and how our thinking and scope evolved over time.

As one of the contributors to the new edition (please note my conflict of interest), I can attest to Miquel Porta’s tireless dedication to this challenging job, constantly revising existing definitions and, mainly, expanding and updating the scope of our discipline by adding a couple of hundred new entries. For example, the COVID-19 pandemic led to detailed discussions of airborne transmission, of superspreading events, and of the infodemic.

Rapid changes in epidemiological thinking, particularly regarding causal inference, have been reflected in consecutive issues of the Dictionary. The fifth edition, the first edited by Miquel Porta, featured a substantial expansion of genetic, molecular, and life-course epidemiological terminology, while the sixth edition focused on visualizing confounding, selection bias, and measurement bias using directed acyclic graphs (DAGs) and on mathematically adjusting for them using g-methods. The current, seventh edition describes the relevance of these new concepts to practical study design. Instead of using DAGs solely for mathematical adjustment of non-randomized studies, target trial emulation (TTE) requires researchers to explicitly design their observational studies to emulate a hypothetical, randomized controlled trial.

Going beyond recent methodological advances, the new edition also reflects how our thinking about health has changed in the post-pandemic era. By expanding the discussion on One Health and Planetary Health, the seventh edition acknowledges that human disease patterns are directly linked to large-scale ecological disruption, including biodiversity loss, wildlife-human interfaces (zoonotic spillovers), and climate change. It also integrates the concept of a syndemic (such as COVID-19 and chronic diseases) with noxious social and environmental interactions. This shifts the focus from looking at diseases in isolation to examining how systemic societal inequities (a major focus of the fifth edition) actively exacerbate biological disease pathways.

As artificial intelligence increasingly shapes how public health-relevant data is synthesized and accessed, the *Dictionary*’s commitment to conceptual precision becomes even more vital. AI models can aggregate information, but they require rigorous, peer-reviewed definitions, as summarized in this volume, to avoid disseminating incorrect concepts. Moving forward, the next editions will surely expand to map the intersections of epidemiological methods with machine learning, algorithmic bias, and climate-related health research. Ultimately, Miquel Porta’s latest edition proves that while our tools change in format—from a checkbook-sized pocket handbook to an e-book on a smartphone—the necessity for clear, rigorous epidemiological thinking remains absolute.

